# Supplementary material for: Fast widefield scan provides tunable and uniform illumination optimizing super-resolution microscopy on large fields
Source: Nat Commun. 2021 May 24;12:3077. doi: 10.1038/s41467-021-23405-4 (PMC8144377; doi:10.1038/s41467-021-23405-4)
Supplement: Supplementary file 1 — Supplementary Information [file 41467_2021_23405_MOESM1_ESM.pdf]

Fast widefield scan provides tunable and uniform illumination optimizing super-resolution microscopy on large fields

Adrien Mau[1,2]*, Karoline Friedl[2,3], Christophe Leterrier[3], Nicolas Bourg[2] and Sandrine Lévêque-Fort[1]*

1 Université Paris-Saclay, CNRS, Institut des Sciences Moléculaires d'Orsay, 91405, Orsay, France.
2 Abbelight, 191 avenue Aristide Briand, Cachan, France
3 Université Aix-Marseille, CNRS, Institut de neurophysiopathologie, Marseille, France
*Corresponding authors: adrien.mau@universite-paris-saclay.fr
sandrine.leveque-fort@universite-paris-saclay.fr

Supplementary Figure 1: Field synthesis with different gaps between scanning lines and different scanning patterns

Supplementary Figure 2: Chronogram of ASTER scanning excitation and camera integration.

Supplementary Figure 3: Vignetting limits the maximum homogeneous FOV

Supplementary Figure 4: Implementation of TIRF and oblique illumination in classical and ASTER excitation schemes

Supplementary Figure 5: Imaging of beads under different illumination parameters

Supplementary Figure 6: Beads for calibrations of optical sectioning

Supplementary Figure 7: Full-field TIRF uniform ASTER excitation at 5ms integration time.

Supplementary Figure 8: Homogeneity of ASTER TIRF excitation compared to azimuthal spinning TIRF

Supplementary Figure 9: Workflow analysis of nanoruler images

Supplementary Figure 10: Gaussian illumination effects in single-molecule STORM microscopy

Supplementary Figure 11: Impact of scanning on STORM blinking properties

Supplementary Figure 12: Stitching of STORM images resulting in a 300 µm x 300 µm field of view

Supplementary Figure 13: Fast STORM experiment acquired at 5ms integration time

Supplementary Figure 14: STORM 200 µm x 200 µm image of neuronal β2-spectrin.

Supplementary Figure 15: STORM 200 µm x 200 µm 3D image of COS-7 cells labeled for microtubules using AF647-coupled antibodies.

Supplementary Notes 1: Comparison of uniform excitation methods

Supplementary Notes 2: Relation between minimum frame rate and field size with ASTER

Supplementary Notes 3: Uncertainties in measurement of microbead excitation depth

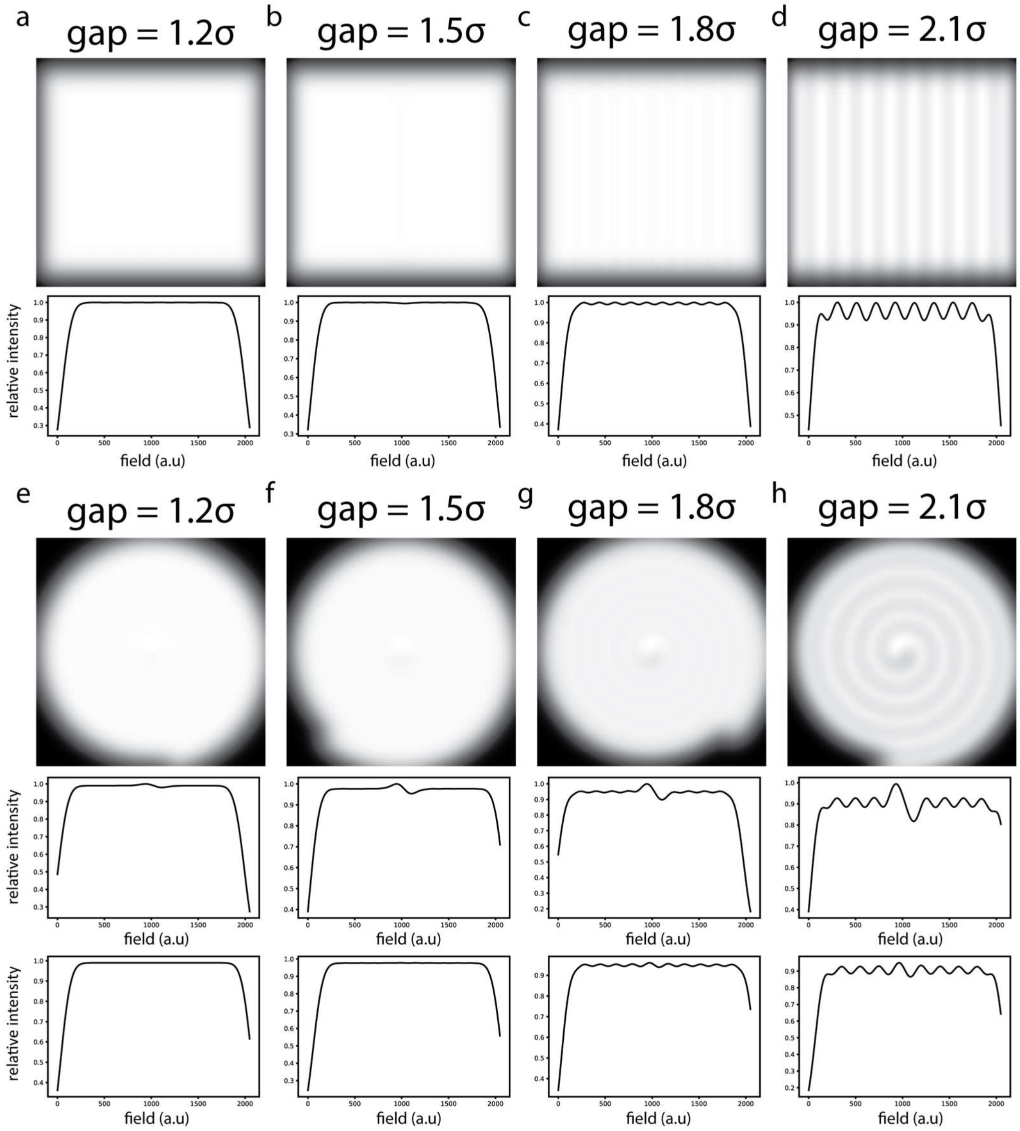

Supplementary Figure 1 Field synthesis with different gaps between scanning lines and different scanning patterns. **(a-d)** Resulting illumination for a raster-scanning pattern and different line gaps. Under each image, resulting horizontal profiles taken at center are shown. **(e-f)** Resulting illumination for scanning an Archimedes spiral at different line gaps. Under each image, resulting illumination profiles along vertical (up) and horizontal (bottom) axes are shown. σ denotes the standard deviation of the scanned Gaussian beam.

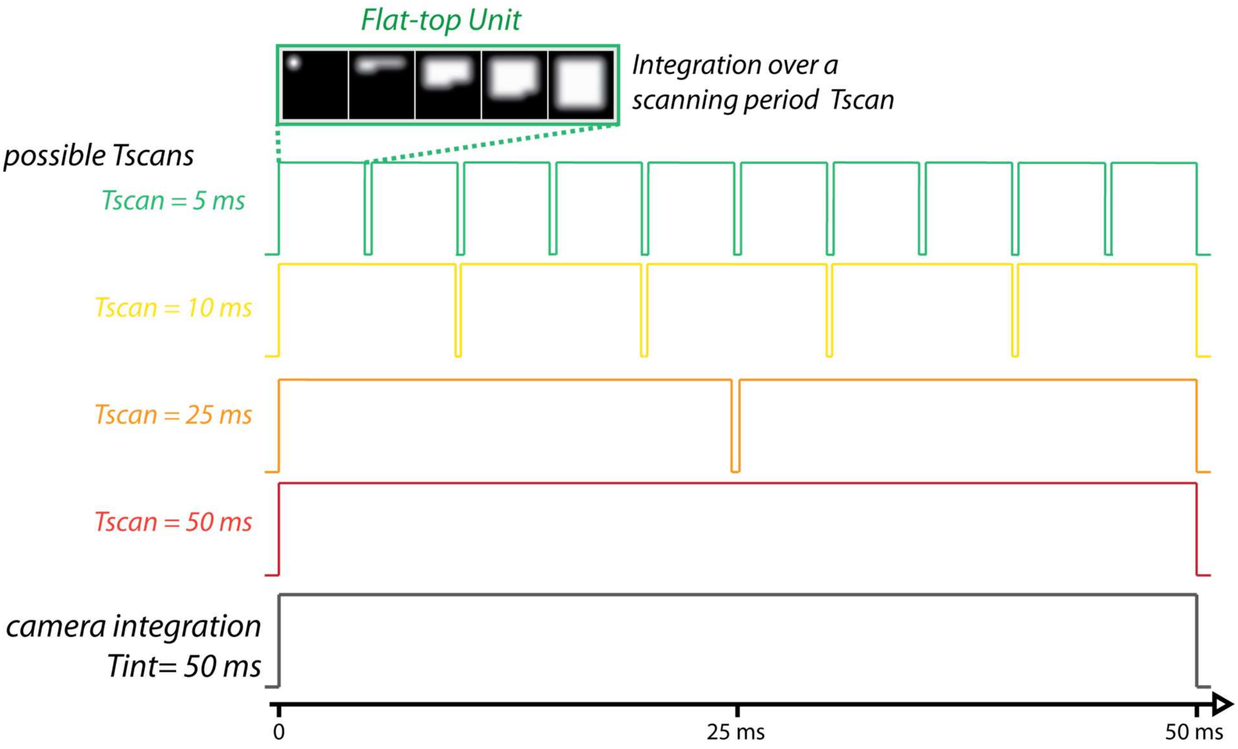

Supplementary Figure 2 Chronogram of ASTER scanning excitation and camera integration. For a given camera integration time (here Tint=50 ms), the scanning period Tscan of ASTER should divide Tint so that a finite number of flat-tops are generated over the integration. Examples for Tscan values are shown, namely 5 ms, 10 ms, 25 ms and 50 ms.

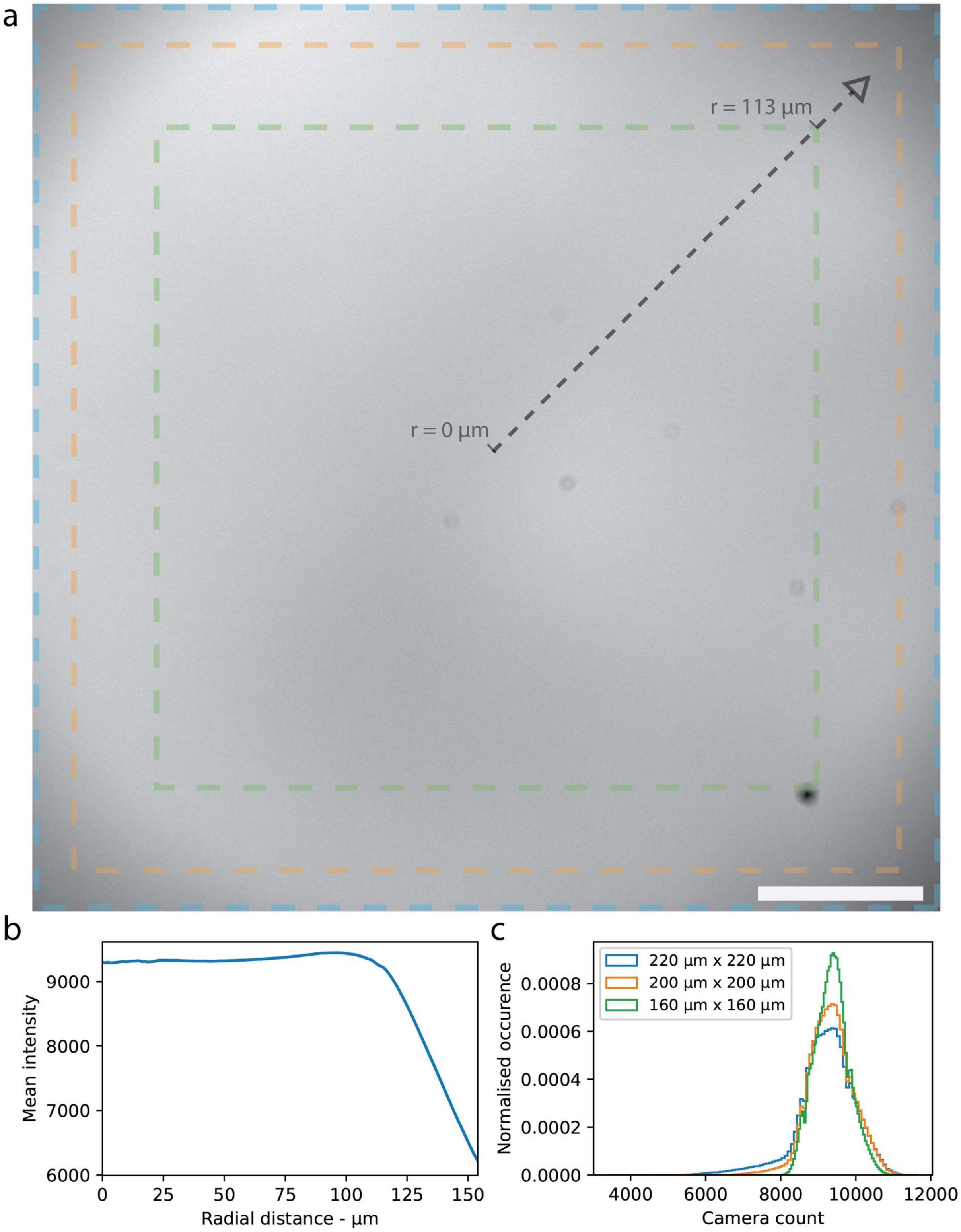

Supplementary Figure 3 Vignetting limits the maximum homogeneous FOV. **(a)** ASTER excitation of a thin layer of Nile-Blue exhibiting fading at the edges. Scalebar, 40 µm. **(b)** Mean intensity at increasing distance from the center. **(c)** Intensity histogram for the full 220 µm x 220 µm field of the camera, a restricted 200 µm x 200 µm and 160 µm x 160 µm fields.

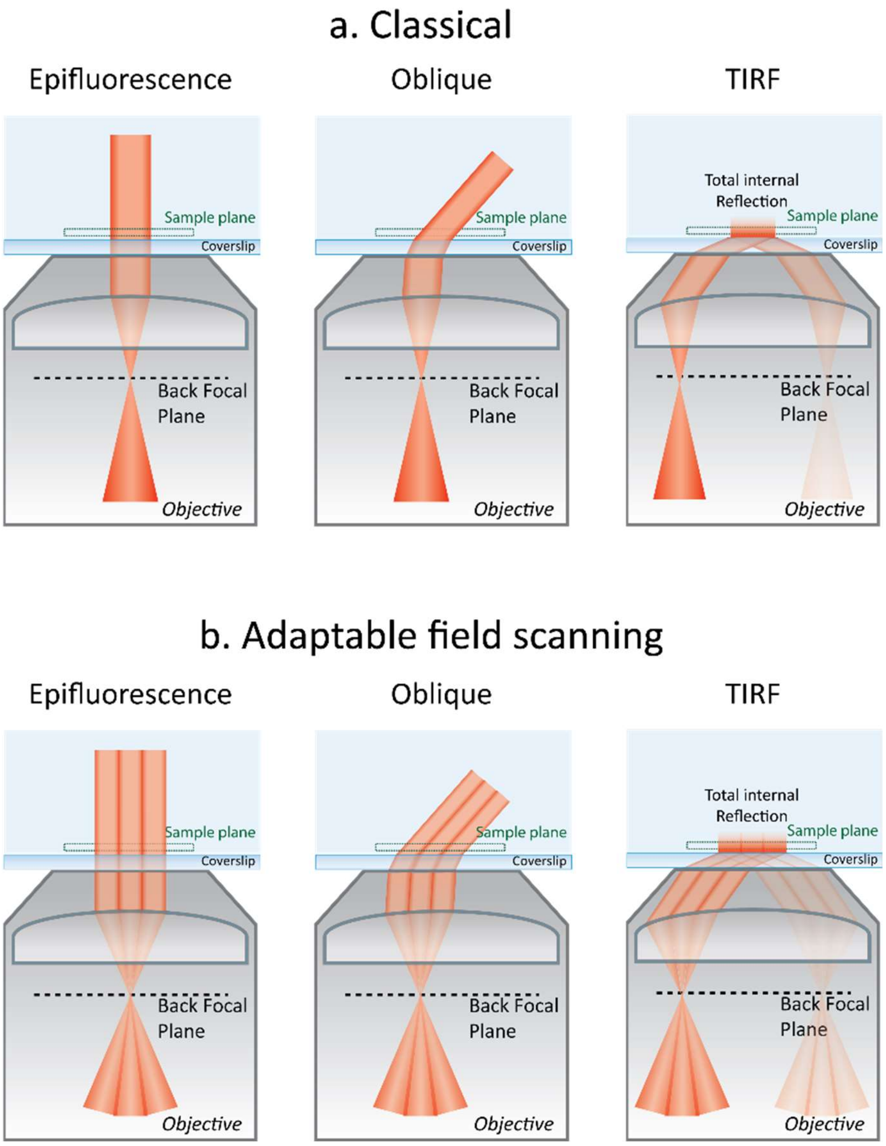

Supplementary Figure 4 Implementation of TIRF and oblique illumination in classical and ASTER excitation schemes. **(a)** Classical configuration in EPI, oblique HiLo and TIRF, from left to right. Each position in the Back Focal Plane coincides with a given output angle. **(b)** ASTER configuration for EPI, oblique HiLo and TIRF, where the scanning effect modifies the effective field of view but does not affect the output angle.

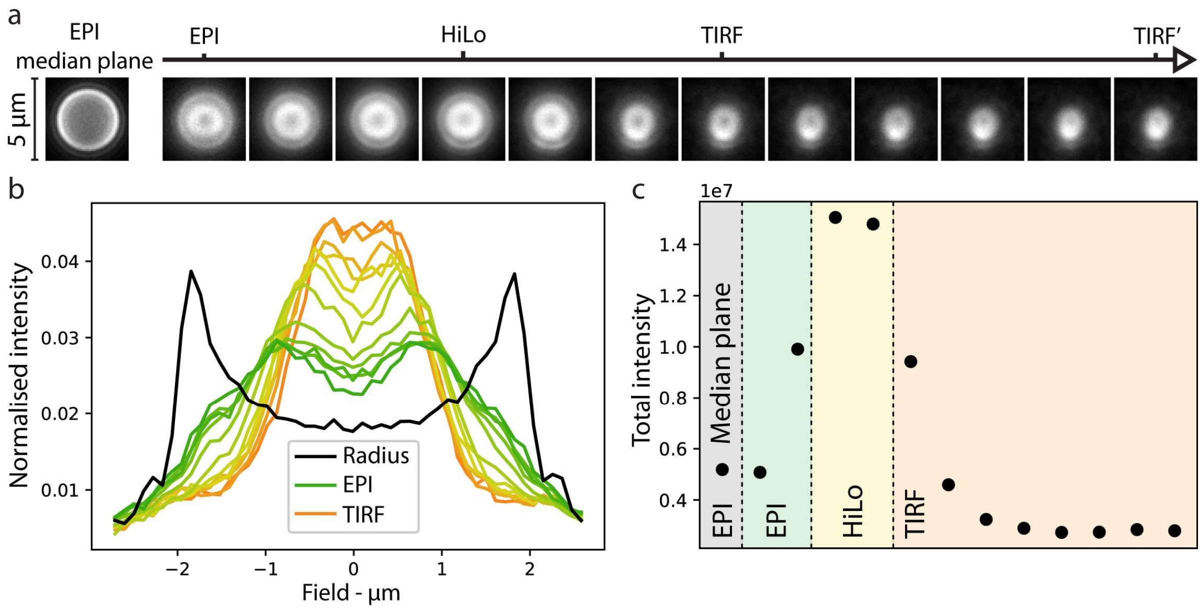

Supplementary Figure 5 Imaging of beads under different illumination parameters **(a)** Imaging of a bead in EPI with focus at median plane (left), and imaging of the same bead with focus at proximity of the coverslip and successive increments of the beam position in the BFP, i.e with an illumination from EPI to TIRF. **(b)** Resulting normalized intensity profiles for the images in (a), 'Radius' indicates the image taken at median plane. **(c)** Total intensity of images in (a). The increase of intensity at HiLo is notable.

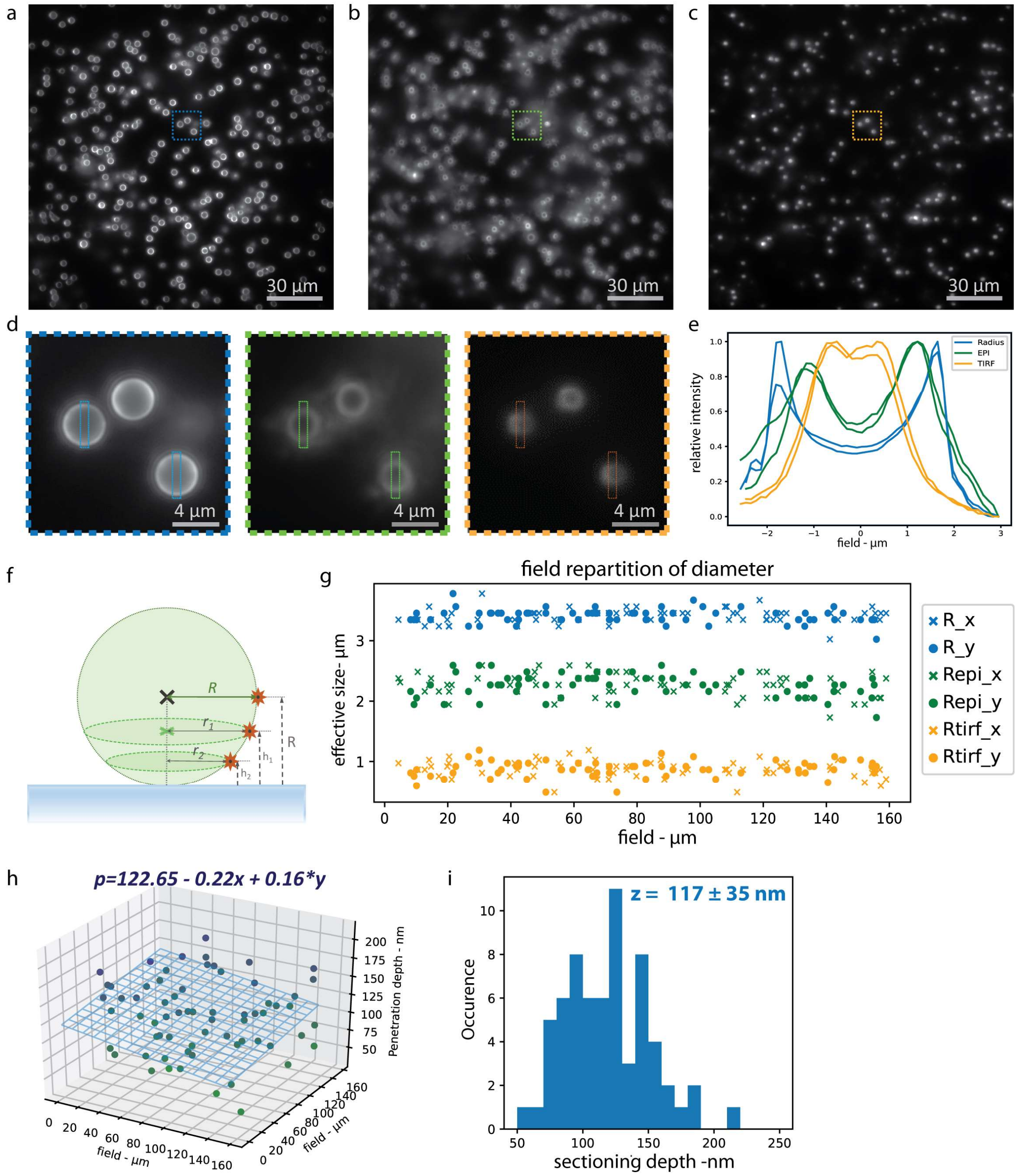

Supplementary Figure 6 Beads for calibrations of optical sectioning. **(a)** Imaging of beads in EPI (classical epifluorescence), with focus at the beads median plane. **(b)** Imaging of beads in EPI, with focus at the coverslip. **(c)** Imaging of beads in TIRF, with focus at the coverslip. **(d)** Close up view of highlighted areas in (a-c) showing that each illumination condition results in its own effective bead radius. **(e)** Vertical profiles of highlighted cross-sections in (d). **(f)** Schematic of a nanobead, with R the median radius, and intermediary radii r_i corresponding to different heights h_i, respecting $R^2 = (R-h_i)^2 + r_i^2$. **(g)** Distribution of the radius measured for each sphere along the field. A cross (respectively a circle) denotes a measurement along the x (respectively y) axis. **(h)** Fitting of the 2D distribution of sectioning depth by a plane (n = 67 spheres). **(i)** Distribution of the TIRF measured sectioning depth.

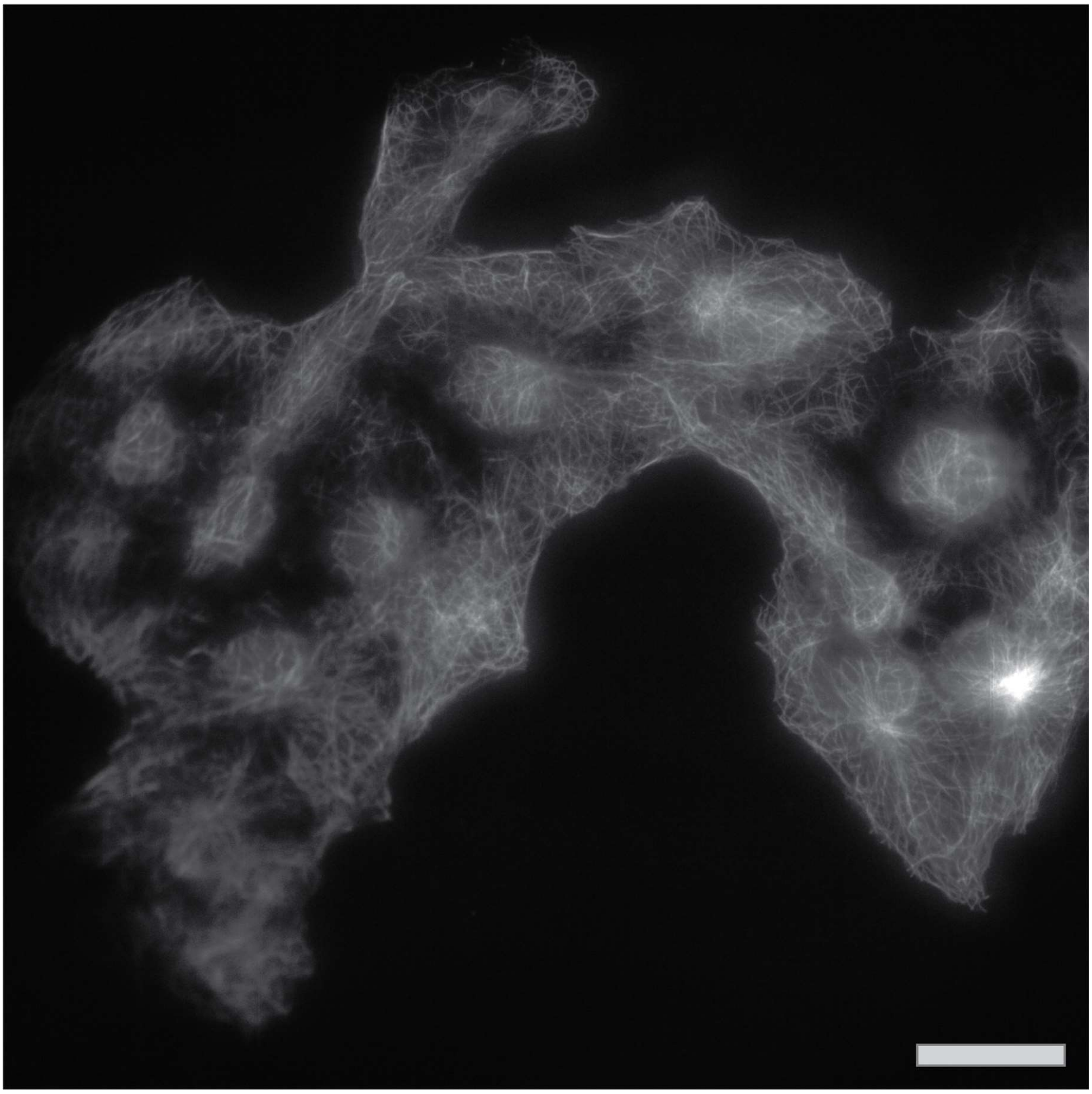

Supplementary Figure 7 Full-field TIRF uniform ASTER excitation at 5 ms integration time. **(a)** Resulting image of COS-7 cells labeled for microtubules using AF647-coupled antibodies imaged over 220 µm x 220 µm. Scanning consisted in twelve lines scanned with a scanning period of 5 ms. Scalebar, 30 µm. This experiment was repeated with similar results on various FOV and biological samples.

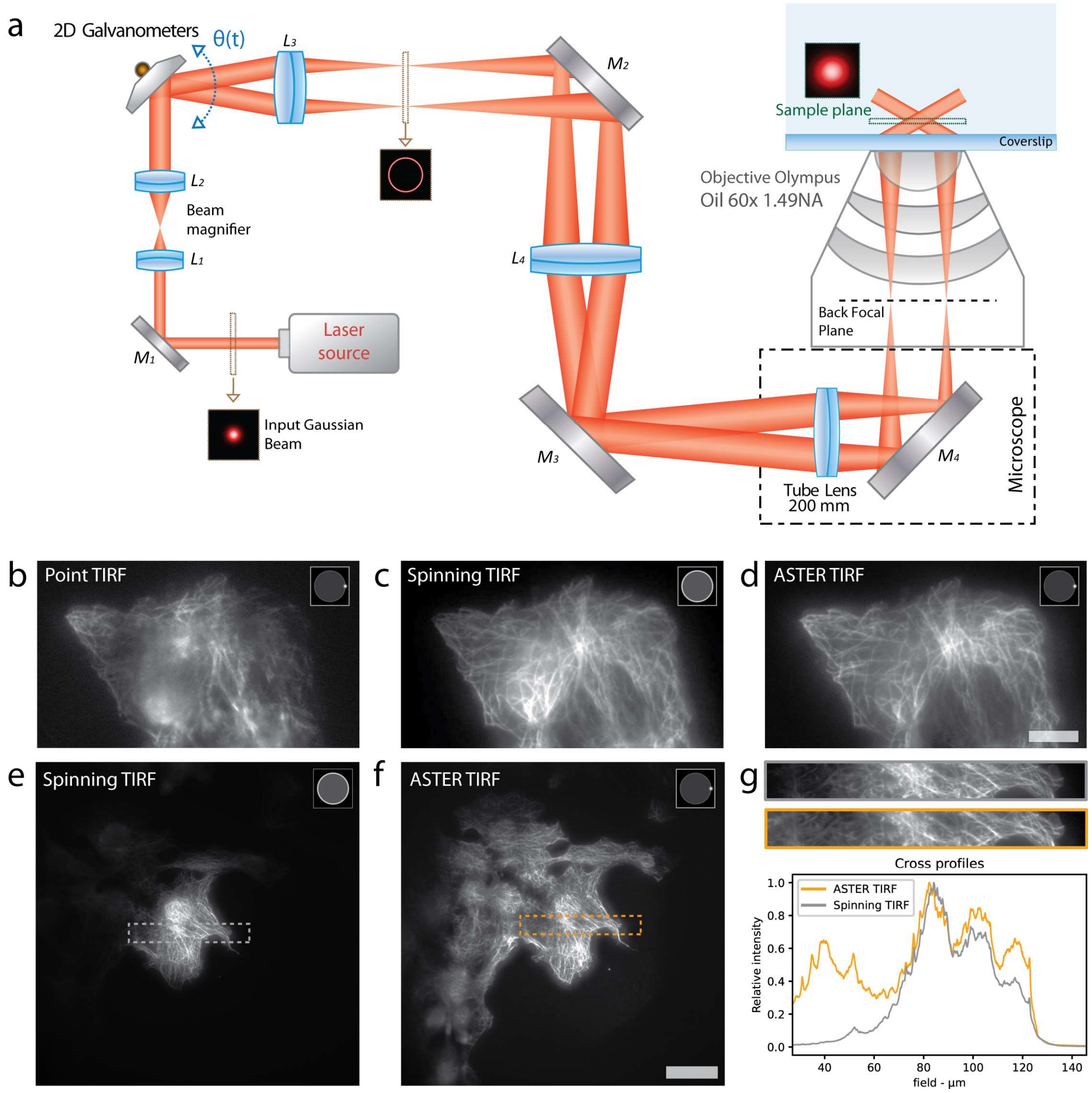

Supplementary Figure 8 Homogeneity of ASTER TIRF excitation compared to azimuthal spinning TIRF. **(a)** Adaptation of ASTER setup to perform azimuthal spinning TIRF by scanning the beam in a sample-conjugated plane. **(b-f)** TIRF images of COS-7 cells labeled for microtubules using AF647-coupled antibodies excited either with an azimuthal spinning setup without scanning (b), with scanning (c,e), or with an ASTER uniform excitation setup (d,f). Notable inhomogeneities in image (b) are not present in images (c) and (d), which exhibit similar TIRF quality. Scalebar, 10 µm. This experiment was repeated thrice and resulted in similar comparison. (e) and (f) are full field images, whose highlighted sections are shown in **(g)**. Compared to ASTER, the gray profile from image (e) show a regular decrease in intensity around 80 µm. Scalebar, 40 µm

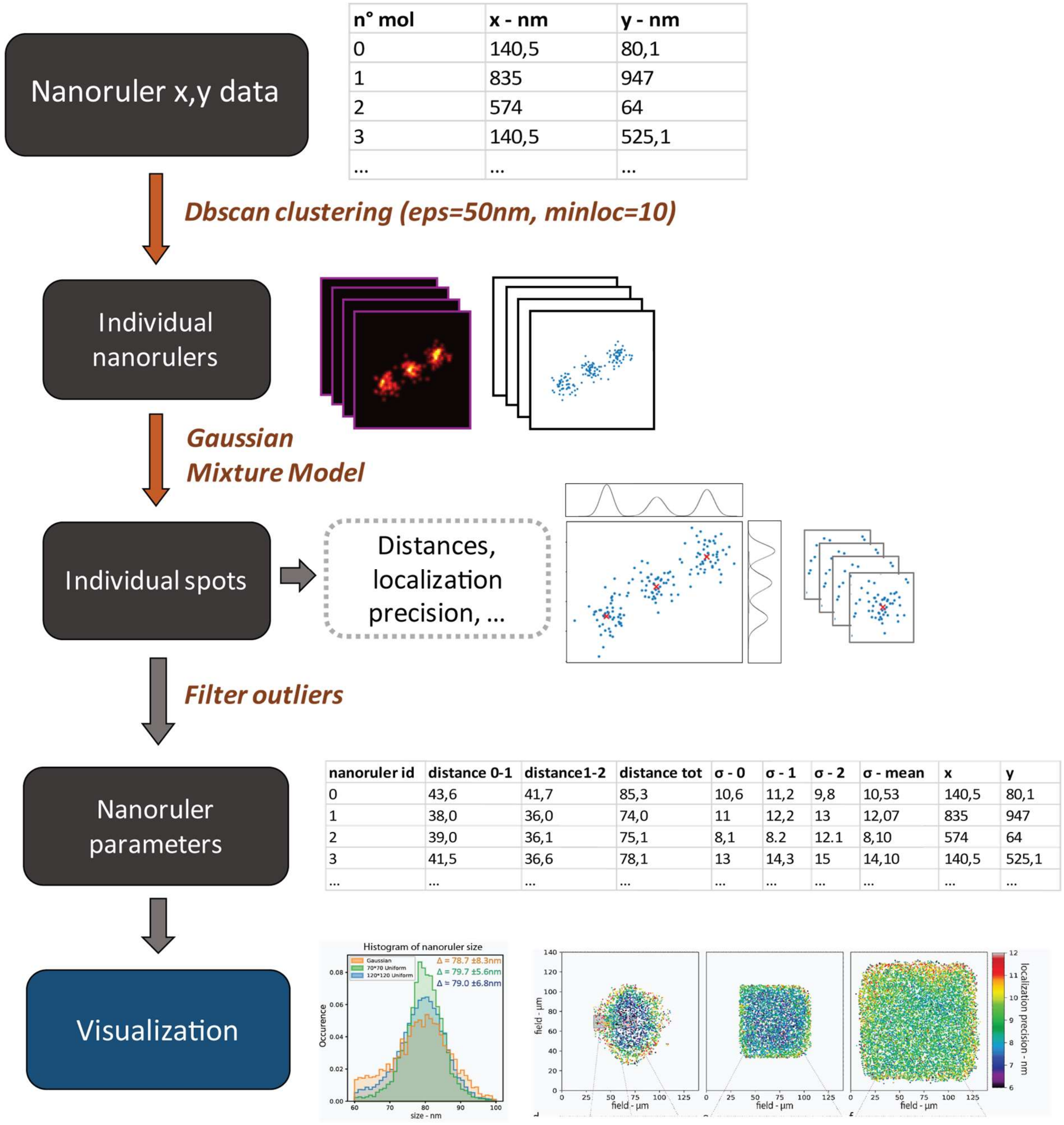

Supplementary Figure 9 Workflow Analysis for three spot nanorulers. (See Methods). From the cloud of localization points, a DBscan isolates each individual nanoruler. One nanoruler consists of three aligned spots, each separated by 40 nm. For each individual nanoruler a Gaussian mixture model fit the localization point clouds by three 2D normal distributions. This estimates which parameters are the most likely to produce the observed point distribution, namely the position and standard deviation of each of the three spots. Each point can then be associated to its most probable spot. This allows for measurement of nanoruler sizes, and estimation of localization precision for all individual nanorulers. Visualization is then performed with the Python library matplotlib.

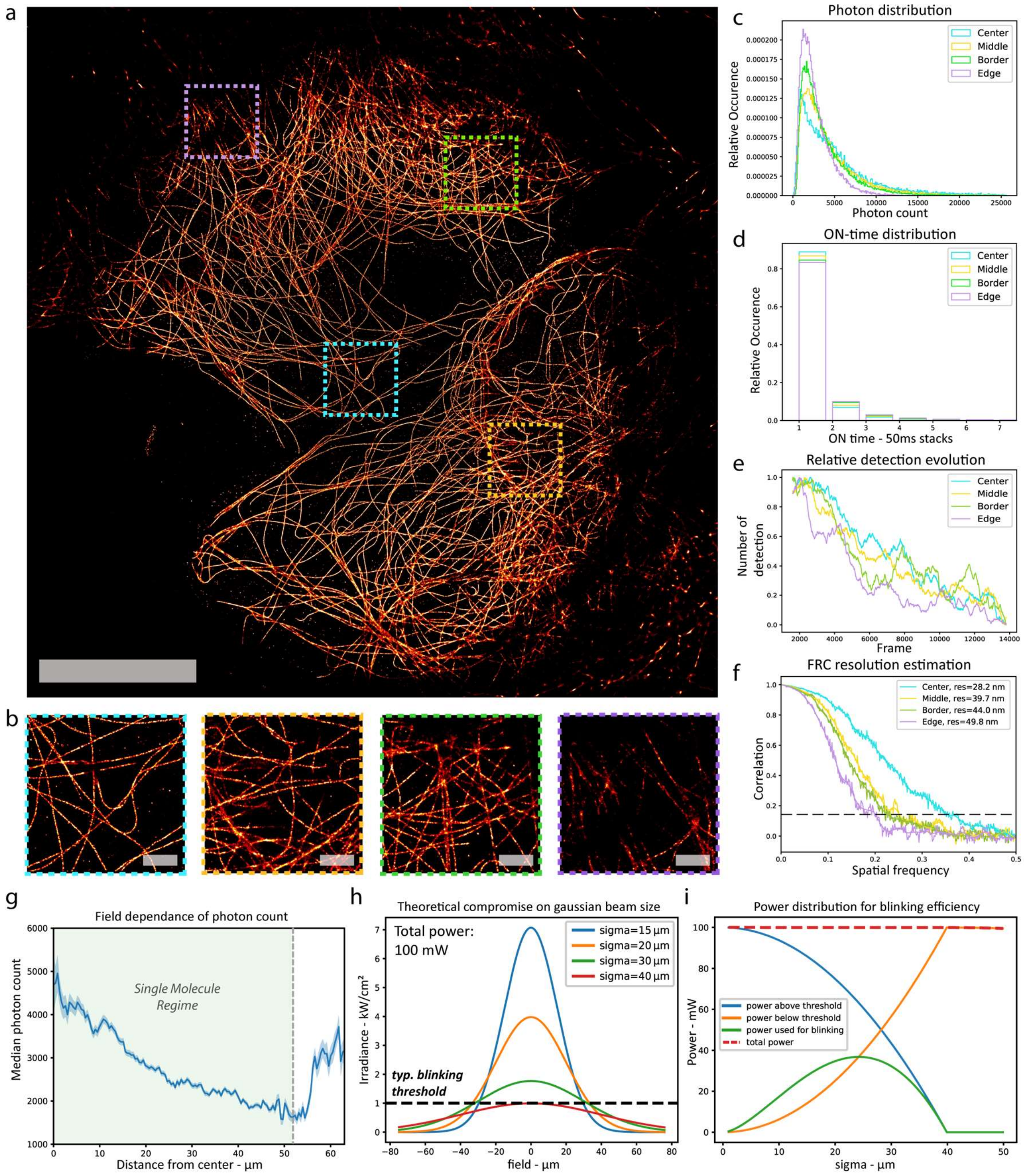

Supplementary Figure 10 Gaussian illumination effects in single molecule STORM microscopy (σ = 45 µm). **(a)** Gaussian STORM imaging of COS-7 cells labeled for microtubules using AF647-coupled antibodies. Detection was done on 20000 frames at 50 ms exposure time with 300 mW laser power. Scalebar, 10 µm. **(b)** Close up views of highlighted areas in (a). Scalebar 1µm. **(c)** Photon count distribution histogram for highlighted areas in (a). **(d)** Blinking ON-time distribution for highlighted areas in (a), expressed in number of frames (50 ms). **(e)** Temporal evolution of detection count for highlighted areas in (a). **(f)** FRC estimation of resolution for highlighted areas in (a). **(g)** Radial median photon count distribution for the total of 11 million localizations. The shaded area indicates the standard deviation above and below the median value. When the single molecule regime is broken, multiple fluorophores are detected and result in overestimation of photon count. **(h)** Compromise on Gaussian beam size to reach a given blinking threshold with a fixed total power of 100 mW. **(i)** Resulting repartition of power usage. Power used for blinking is the product of the blinking threshold by the area that is illuminated over this threshold.

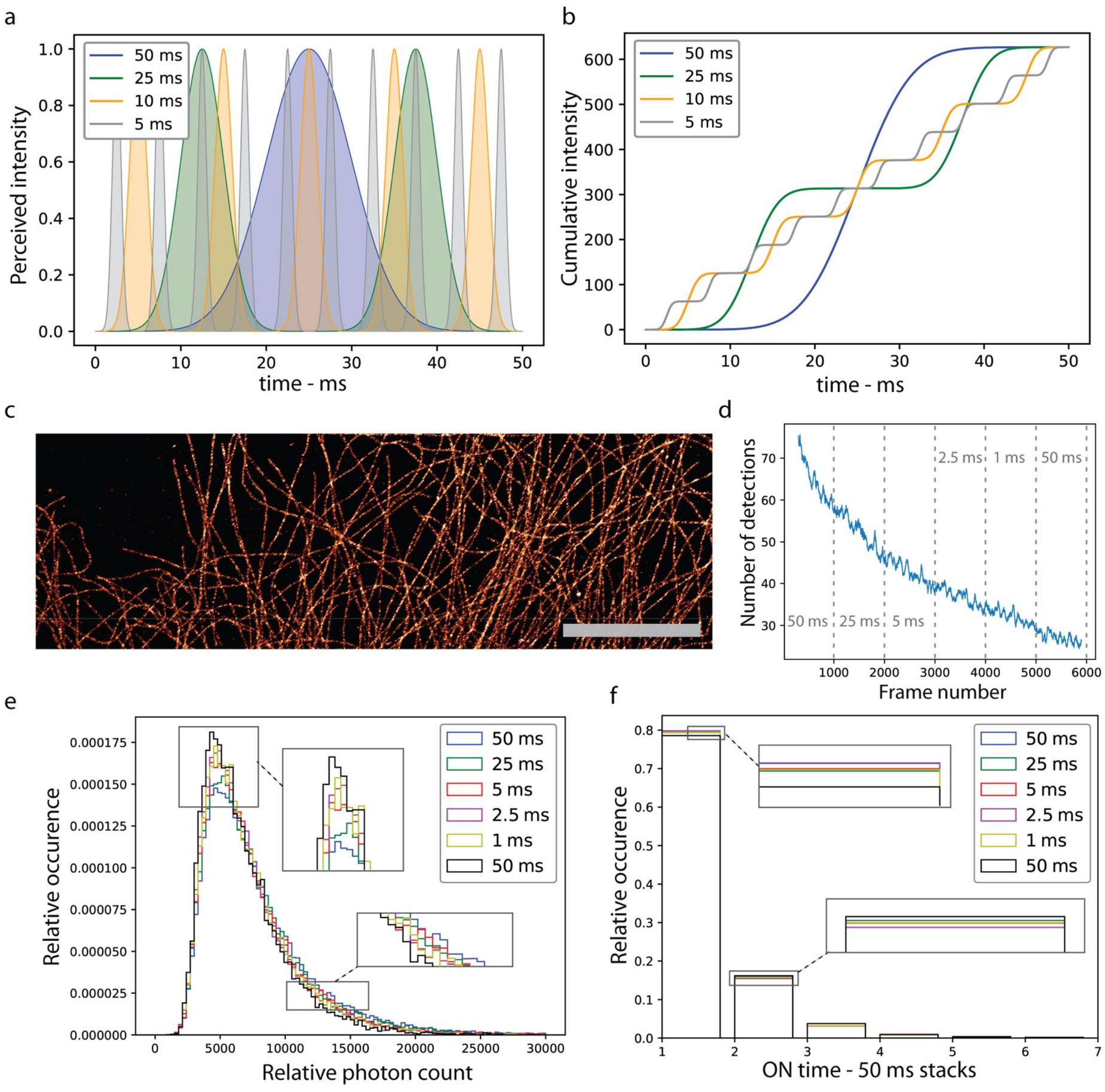

Supplementary Figure 11 Impact of scanning on STORM blinking properties. **(a)** Perceived irradiance along time for a fluorophore excited under different ASTER scanning periods. **(b)** Cumulative intensity for each scanning period over 50 ms, resulting in a similar mean irradiance. **(c-f)** STORM imaging of COS-7 cells labeled for microtubules using AF647-coupled antibodies over a constant field with varying scanning periods. Scanning period is modified every 1000 frames and chronologically takes values 50, 25, 5, 2.5, 1 and 50 ms. (c) Resulting image over the whole frames. Scalebar, 10 µm. (d) Number of molecules detected per frame. Gray text indicates the experimental scanning speed for each frame range. (e) Relative photon count histogram for each scanning speed. (f) Relative ON time histogram for each scanning speed.

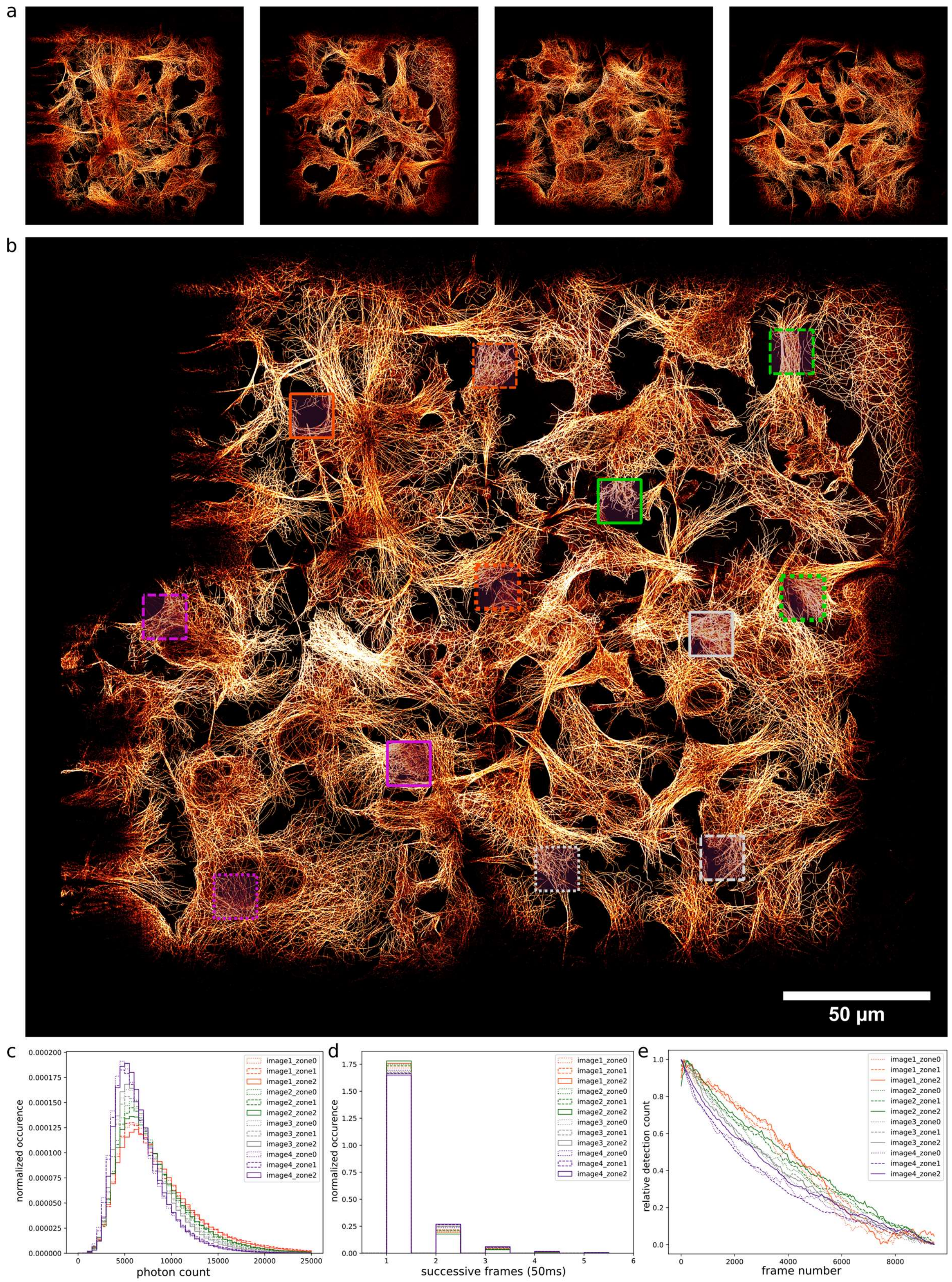

Supplementary Figure 12 Stitching of four STORM images resulting in a 300 µm x 300 µm field of view. **(a)** Individual 160 µm x 160 µm STORM images of COS-7 labeled for microtubules. **(b)** Stitched images resulting in a 300 µm x 300 µm field. **(c-e)** Normalized photon count histograms (c), normalized ON time histograms (d) and relative number of detections along frames (e) for highlighted areas in (b).

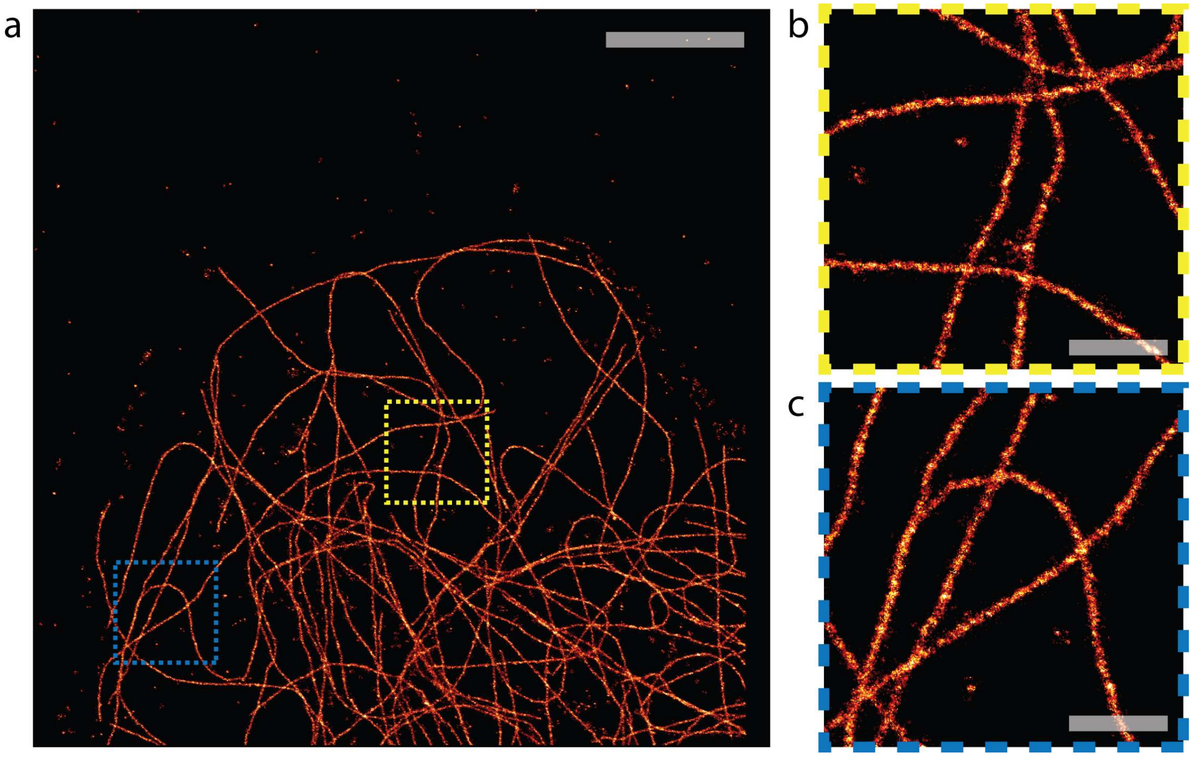

Supplementary Figure 13 Fast STORM experiment acquired at 5ms integration time. Instantaneous irradiance of 24 kW/cm² **(a)** Resulting 10 nm-pixel image of COS-7 cells labeled for microtubules using AF647-coupled antibodies for a 20000 frames (100 seconds) acquisition. After filtering outliers, 650 000 molecules contribute to the final image with an average density of 1000 molecules/µm². Scalebar, 5 µm. **(b-c)** Close up views of highlighted areas in (a). Scalebar, 1 µm.

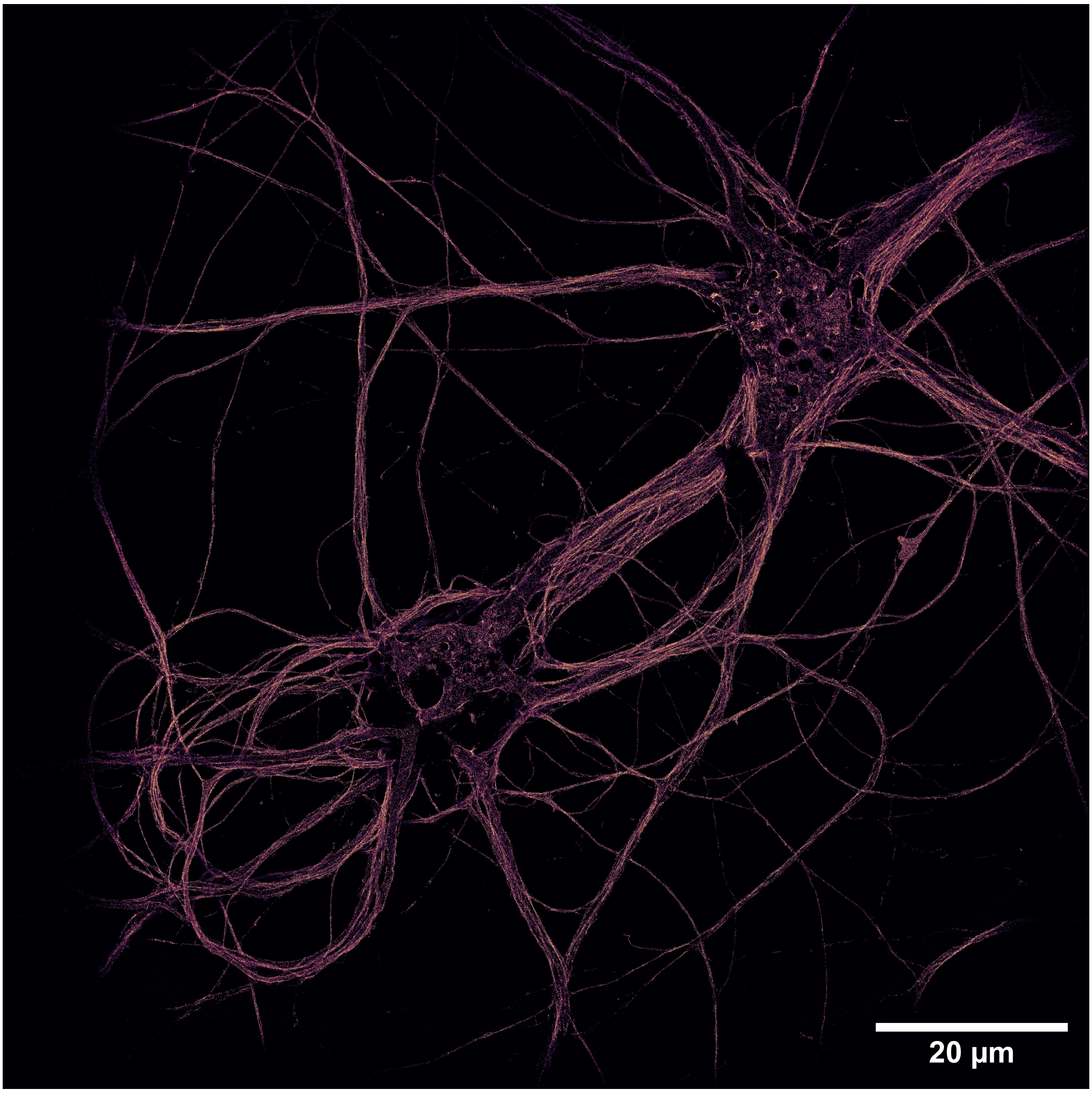

Supplementary Figure 14 STORM 200 μm x 200 μm image of neuronal β2-spectrin, labeled with AF647. Similar large field images were obtained for multiple acquisitions (n=5).

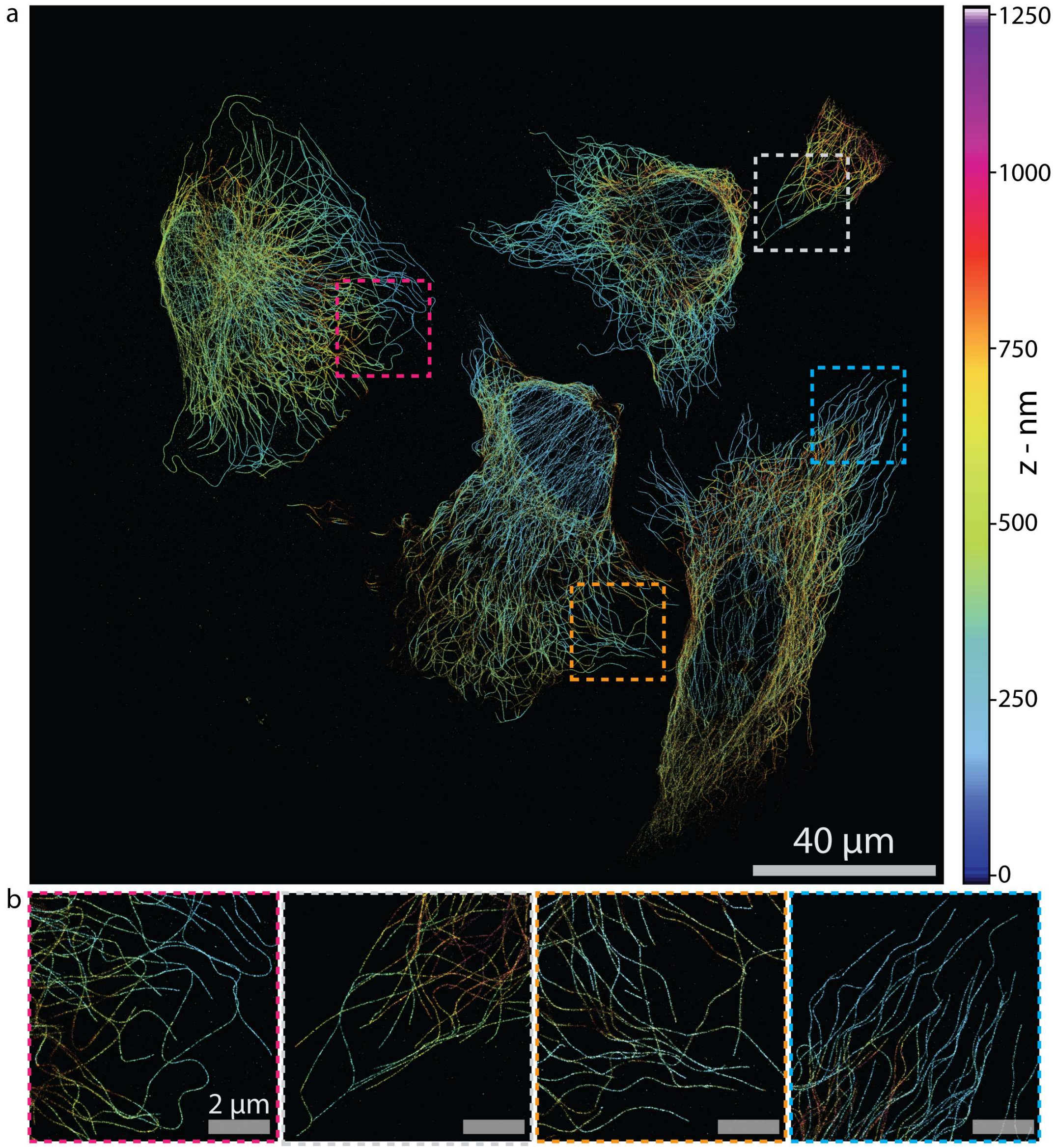

Supplementary Figure 15: STORM 200 µm x 200 µm 3D image of COS-7 cells labeled for microtubules using AF647-coupled antibodies. **(a)** 3D color encoded image of microtubules, reconstruction pixel is 40nm and a 1 pixel Gaussian blur has been applied. Axial information was obtained by splitting the detection relay in two paths and placing a 16k astigmatic lens on one path. **(b)** 20 µm x 20 µm images of highlighted areas in (a), with a reconstruction pixel of 10 nm. Experiment was repeated thrice with similar results.

Supplementary Notes 1: Comparison of uniform excitation methods

There are many ways to provide a uniform illumination[1], which can be classified in two general categories, and compared with ASTER:

- Waveguides: Waveguides provide uniformity on extremely large FOVs[2,3], but are impossible to adapt as they provide a fixed excitation depth, and a fixed illumination size. They illuminate the sample with evanescent waves and are adapted to experiments that need low power and optical sectioning restrained to the proximity of the coverslip, as in the case of PAINT experiments[4]. They are relatively low cost and achromatic, but can be complex to implement as mounting of the sample and coupling of the laser is not performed in a classical way.

- Direct beam reshapers: Beam reshapers directly transform a Gaussian propagative beam into a flat-top. This includes optical beam-shapers[5–7] (such as those from Pishaper, Topshape or Topag), square core fibers[8–10]; microlens-array[11,12] and phase SLM[13]. Due to the nature of light, the flat-top profile is not maintained along its propagation and care must be given to the optical alignment of optical planes, so that the top-hat yields optimal contrast at sample plane. Beam reshapers are relatively achromatic, and with the addition of a translation stage allow shifting of the output illumination angle to perform optical sectioning techniques. A primary drawback of direct beam-reshapers is that they are ill-adapted to quantitative TIRF: in the back focal plane, the flat-top profile should approximately take the shape of its Fourier transform, resulting in a sinc shape. Even though qualitative TIRF is achievable it should be hampered by multiple output angles, as it is not possible to precisely restrain the width of the beam in the back focal plane. Furthermore, such system may suffer from speckle patterns. A rotating diffuser or vibrating membranes can be placed on the path of the beam and will typically average out the speckle in the order of 10 ms. When using fibers however, it is possible to use a vibrating motor to average the speckle in less than 1 ms, but will waste more laser power than beam-shapers elements as coupling in the fiber will typically waste 40% of the input power. It can be balanced by using multiple lasers with beam combiners[10], but will increase the system complexity and cost. In term of FOV size, beam reshapers can always be supplemented with an afocal system, so that even if the input and/or output beam is restrained in size, it will not be limiting in the global illumination setup, but once a size has been chosen it will be complex to change. While optical beam shapers and fibers will require physical intervention to adapt the flat-top size, the microlens-array device can slightly adapt the illumination by moving its components. On this matter, piSMLM[13], which uses a phase only SLM to shape the beam is extremely adaptable, but also results in great power losses (~90%).

- ASTER: ASTER is a hybrid scanning-wide-field illumination setup and is close in performance to optical beam-reshapers. It continuously scans a Gaussian beam following specific patterns to provide a flat-top in a time-averaged manner. With the

galvanometer technology, one line can typically be scanned in 300µs, so when using the technology at its limit 16 lines can be scanned in 5 ms to generate a flat-top on wide fields. This high number of lines is compatible with the use of a small base beam of around 8 µm and a spacing of 1.7σ, resulting in a high contrast flat-top profile on 220 µm x 220 µm. Furthermore, even though a flat-top is synthetized, the beam remains Gaussian at each instant and the synthetized field keeps its properties along propagation. In particular, TIRF should not be hampered given that the scanning device is well conjugated to the back focal plane of the objective, where the beam will take the shape of a focalized Gaussian.

No speckle pattern was observed when illuminating with ASTER in EPI, HiLo or TIRF configurations. This is mostly attributed to the scanning effect, that means out the speckle as it simultaneously generates the flat-top and thus gives comparable results to azimuthal spinning TIRF[14].
ASTER is not restrained in input beam size and can easily generate variable excitation sizes and positions. As it is controlled electronically, it can shift from one configuration to another in milliseconds. In that regard, ASTER is the most versatile of all methods, but comes with a complexity in time dependence. The scanning pattern should always be adapted so that the synthesis of uniform illumination does not yield unwanted stroboscopic effects. Assuring that the period of the flat-top synthesis is two times less than the period of the observed phenomena should allow for confident observation.

All of these properties are qualitatively synthetized in this table:

General Scheme	Adaptability	Sectioning	Power Efficiency	Lowest image rate without speckle	Cost of devices	Ease of implementation
**Waveguide**[2–4]	poor	great, fixed	-	not limited	low	Sample handling, coupling in waveguide
**Multimode fiber**[8–10]	physical intervention	average	average	fast motor - 0.5ms	0.2 k€ *	Fiber coupling
**Optical Beam Shaper**[5–7]	physical intervention	average	good	diffuser - several ms	0.5-5 k€	-
**Microlens array**[11,12]	average	not shown	good	diffuser - several ms	0.6k€	Alignment
**phase SLM**[13]	excellent	not shown	poor	diffuser - several ms	10-15 k€	Alignment, control of SLM, calibration
**ASTER**	excellent	good	good	2ms - 5ms (FOV dependant)	1-5 k€	Alignment, electronic control

*actual cost for multimode fiber is higher when considering multiple lasers, beam couplers and rotating motor.

Supplementary Notes 2: Relation between minimum frame rate and field size with ASTER

ASTER relies on the continuous scanning of a wide beam to generate a temporally-averaged flat-top profile. The minimum time needed to generate this profile depends on the size *σ* of the initial beam, and on the resulting side length *D* of the field.

As can be seen in Supplementary Figure 1, in order to generate a flat illumination the minimum distance between close lines must be less than 1.7*σ*. In this article we chose a minimal spacing of 1.2*σ*. Notably, the flat-top effect is maintained even when this length is diminished and scanning close lines should not impact fluorophore blinking (see Supplementary Fig. 11). A small gap however allows us to keep a flat-top profile even when reducing the size of the initial Gaussian beam.

Considering the fact than the step response of the galvanometers is around 300 µs, a line can be scanned in at least 300 µs. We then can estimate the minimum time necessary to generate a field of length *D.* For example, at least 11 lines are needed to generate a uniform field of size *D = 12σ*, resulting in a 3.6 ms minimum generation time. The general formula is:

$$n_{minline} = 1 + ceil(\frac{D}{1.7\sigma})$$

$$T_{min} = n_{minline} * 300\mu s$$

Where $n_{minline}$ is the minimum number of line needed to achieve homogeneity and Tmin the minimum time required to generate the profile. Notably, as we do not perform a point by point scanning but line scans this relation is not proportional to the area of the field but to its shortest length. This formula also shows that the wider the size of the initial beam, the faster we can generate a flat-top. However, the border of the flat-top will exhibit similar shape than that of the initial scanned beam, so that contrast will be hampered if the initial beam is chosen too wide.

In this publication, the initial beam size is 17 µm. Fields of 200 µm x 200 µm can then be generated by scanning 10 lines with a gap of 1.4*σ* which can theoretically be achieved in 3 ms. Smaller fields, such as 30 µm x 30 µm can be achieved with our beam size by scanning 3 lines in less than a millisecond.

In general for SMLM, integration times in the order of 20-100 ms are used, so there is no need to achieve the fastest scanning speed. Instead, the period of the field synthesis should be around two to four times less that of the integration time, this will be enough to guarantee a correct mean flat-top and maintain the galvanometers in a favorable regime.

Supplementary Notes 3: Uncertainties in measurement of microbead excitation depth

Calibration of the TIRF penetration depth among microbeads yields a 117 nm mean value with a standard deviation of 35 nm (Supplementary Figure 6). This arises from the calculus of radii, where we found a standard deviation of 0.11 µm for the median radius, and 0.14 µm for the TIRF effective radius. This radius deviation corresponds to a precision of approximately one pixel (108 nm) and partly reflects the profiles that are represented in Supplementary Figure 6.e, where some peaks cannot be confidently attributed to one of two adjacent pixels. It is plausible that a smaller pixel size would improve precision. On the other hand, assuming that we regularly miss the position of peaks by half a pixel, the deviation should theoretically be around 54 nm. That we still found 0.11 µm -0.14 µm deviation is likely due to other sources of variation: there might be flattening of some microbeads, inhomogeneities occuring at the coverslip and/or a spatial tilting of the sample.

We fitted the measured sectioning depth along the field by a plane and found that a 1 µm increase in the x direction (respectively y) shifted the measured mean sectioning depth by -0.22 nm (respectively 0.16 nm). When taking this tilt into account, the standard deviation of sectioning depths would result in 33.0 nm, which indicates that this tilt does not greatly contribute to the deviation. The tilt could be indicative of either a tilted coverslip, or a slight misalignment of the galvanometers with the back focal plane of the objective. However, in the latter case, a dependence between field and penetration depth would arise preferentially in the direction of the incidence angle (namely x), but not in both directions.

Finally, we checked for local spatial correlation by calculating Moran I and Geary C indexes. Theses indexes characterize the spatial correlation between measurements by using weights as indicators of proximity. For Moran I index, positive spatial correlation (respectively negative) is indicated by a value close to 1 (respectively -1). For Geary C index, positive spatial correlation (respectively negative) is indicated by a value close to 0 (respectively 2).

$$Moran\ I = \frac{N}{\sum_i \sum_j w_{ij}} \cdot \frac{\sum_i \sum_j w_{ij}\,(X_i - \bar{X})(X_j - \bar{X})}{\sum_i (X_i - \bar{X})^2}$$

$$Geary\ C = \frac{N-1}{\sum_i \sum_j w_{ij}} \cdot \frac{\sum_i \sum_j w_{ij}\,(X_i - X_j)^2}{2}$$

The choice of the weight factors *wij* will determine the final results. In order to conclude objectively we calculate Moran and Geary indexes using both boolean and continuous weights. Boolean weights *wij* equal 1 if and only if beads *i* and *j* are the closest neighbors, while continuous weights are based on distances and increase with proximity between beads.

	**Coefficients**	
**Indexes**	Continuous	Booleans
Moran I	-0.004	0.245
Geary C	0.996	0.843

Table 1 : Calculated Moran I and Geary C indexes for n=66 microbeads from Figure 3. Weights were ether chosen continuously with distances or as Booleans with closest neighbour.

As can be assessed on Table 1, values of indexes are close to 0 for Moran, and 1 for Geary C indexes, no matter the choice of weights. This is indicative of a random spatial distribution at the scale of the microbeads. We conclude that there is no strong spatial correlation between close

beads (micrometers apart) and their measured penetration depth. Though local inhomogeneities may exist at smaller scales, the effective optical sectioning of our experiment can be considered globally uniform.

References:

1. Ibrahim, K. A., Mahecic, D. & Manley, S. Characterization of flat-fielding systems for quantitative microscopy. *Opt. Express* **28**, 22036 (2020).

2. Diekmann, R. *et al.* Chip-based wide field-of-view nanoscopy. *Nat. Photonics* **11**, 322–328 (2017).

3. Ramachandran, S., Cohen, D. A., Quist, A. P. & Lal, R. High performance, LED powered, waveguide based total internal reflection microscopy. *Sci. Rep.* **3**, 2133 (2013).

4. Archetti, A. *et al.* Waveguide-PAINT offers an open platform for large field-of-view super-resolution imaging. *Nat. Commun.* **10**, 1267 (2019).

5. Schreiber, B., Elsayad, K. & Heinze, K. G. Axicon-based Bessel beams for flat-field illumination in total internal reflection fluorescence microscopy. *Opt. Lett.* **42**, 3880 (2017).

6. Stehr, F., Stein, J., Schueder, F., Schwille, P. & Jungmann, R. Flat-top TIRF illumination boosts DNA-PAINT imaging and quantification. *Nat. Commun.* **10**, 1268 (2019).

7. Rowlands, C. J., Ströhl, F., Ramirez, P. P. V., Scherer, K. M. & Kaminski, C. F. Flat-Field Super-Resolution Localization Microscopy with a Low-Cost Refractive Beam-Shaping Element. *Sci. Rep.* **8**, 5630 (2018).

8. Kwakwa, K. *et al.* easySTORM: a robust, lower-cost approach to localisation and TIRF microscopy. *J. Biophotonics* **9**, 948–957 (2016).

9. Deschamps, J., Rowald, A. & Ries, J. Efficient homogeneous illumination and optical sectioning for quantitative single-molecule localization microscopy. *Opt. Express* **24**, 28080 (2016).

10. Zhao, Z., Xin, B., Li, L. & Huang, Z.-L. High-power homogeneous illumination for super-resolution localization microscopy with large field-of-view. *Opt. Express* **25**, 13382–13395 (2017).

11. Coumans, F. A. W., van der Pol, E. & Terstappen, L. W. M. M. Flat-top illumination profile in an epifluorescence microscope by dual microlens arrays. *Cytometry A* **81A**, 324–331 (2012).

12. Douglass, K. M., Sieben, C., Archetti, A., Lambert, A. & Manley, S. Super-resolution imaging of multiple cells by optimized flat-field epi-illumination. *Nat. Photonics* **10**, 705–708 (2016).

13. Chen, S.-Y., Bestvater, F., Schaufler, W., Heintzmann, R. & Cremer, C. Patterned illumination single molecule localization microscopy (piSMLM): user defined blinking regions of interest. *Opt. Express* **26**, 30009 (2018).

14. Mattheyses, A. L., Shaw, K. & Axelrod, D. Effective elimination of laser interference fringing in fluorescence microscopy by spinning azimuthal incidence angle. *Microsc. Res. Tech.* **69**, 642–647 (2006).
