# Supplementary material for: Fast widefield scan provides tunable and uniform illumination optimizing super-resolution microscopy on large fields
Source: Nat Commun. 2021 May 24;12:3077. doi: 10.1038/s41467-021-23405-4 (PMC8144377; doi:10.1038/s41467-021-23405-4)
Supplement: Supplementary file 3 — Reporting Summary [file 41467_2021_23405_MOESM3_ESM.pdf]

nature research

Corresponding author(s): Sandrine LEVEQUE-FORT

Last updated by author(s): 31/03/2021

Reporting Summary

Nature Research wishes to improve the reproducibility of the work that we publish. This form provides structure for consistency and transparency in reporting. For further information on Nature Research policies, see our Editorial Policies and the Editorial Policy Checklist.

Statistics

For all statistical analyses, confirm that the following items are present in the figure legend, table legend, main text, or Methods section.

n/a	Confirmed	
☐	☒	The exact sample size (n) for each experimental group/condition, given as a discrete number and unit of measurement
☐	☒	A statement on whether measurements were taken from distinct samples or whether the same sample was measured repeatedly
☒	☐	The statistical test(s) used AND whether they are one- or two-sided<br>*Only common tests should be described solely by name; describe more complex techniques in the Methods section.*
☒	☐	A description of all covariates tested
☒	☐	A description of any assumptions or corrections, such as tests of normality and adjustment for multiple comparisons
☐	☒	A full description of the statistical parameters including central tendency (e.g. means) or other basic estimates (e.g. regression coefficient) AND variation (e.g. standard deviation) or associated estimates of uncertainty (e.g. confidence intervals)
☒	☐	For null hypothesis testing, the test statistic (e.g. F, t, r) with confidence intervals, effect sizes, degrees of freedom and P value noted<br>*Give P values as exact values whenever suitable.*
☒	☐	For Bayesian analysis, information on the choice of priors and Markov chain Monte Carlo settings
☒	☐	For hierarchical and complex designs, identification of the appropriate level for tests and full reporting of outcomes
☒	☐	Estimates of effect sizes (e.g. Cohen's d, Pearson's r), indicating how they were calculated

Our web collection on statistics for biologists contains articles on many of the points above.

Software and code

Policy information about availability of computer code

Data collection	Neo-Live (Abbelight) was used for image acquisition
Data analysis	Single molecule analysis was performed either with a homemade Python 3.7 code (available online https://github.com/AdrienMau/ASTER_code) or with Neo-Analysis (Abbelight).

For manuscripts utilizing custom algorithms or software that are central to the research but not yet described in published literature, software must be made available to editors and reviewers. We strongly encourage code deposition in a community repository (e.g. GitHub). See the Nature Research guidelines for submitting code & software for further information.

Data

Policy information about availability of data

All manuscripts must include a data availability statement. This statement should provide the following information, where applicable:

- Accession codes, unique identifiers, or web links for publicly available datasets
- A list of figures that have associated raw data
- A description of any restrictions on data availability

Data availability is provided : SMLM large data files (>20Go) are available from the corresponding author on reasonable request. Other data files are available on Zenodo (DOI: 10.5281/zenodo.3814322)

Field-specific reporting

Please select the one below that is the best fit for your research. If you are not sure, read the appropriate sections before making your selection.

- [x] Life sciences
- [ ] Behavioural & social sciences
- [ ] Ecological, evolutionary & environmental sciences

For a reference copy of the document with all sections, see nature.com/documents/nr-reporting-summary-flat.pdf

Life sciences study design

All studies must disclose on these points even when the disclosure is negative.

Sample size

67 Beads were used for measurement of the optical sectioning in TIRF. Nano-rulers : 2393, 4876 and 11822 nanorulers were respectively used for the Gaussian, the 70 µm x 70 µm and the 120 µm x 120 µm ASTER illuminations. For clathrin clustering, 20285 clathrin structures were used.

Data exclusions

No data excluded.

Replication

Experiments with Nile Blue have been replicated five times, yielding similar results. When investigating vignetting, full field images of Nile-Blue samples have been replicated three times.
The effect of optical sectioning on beads was observed multiple times (n>5) on three different samples. Optical imaging from EPI to TIRF, of neurons or other biological component, is highly consistent : it has been replicated multiple times (n>20) on different setups. The ability of our implementation to perform TIRF images with a scanning period of 5ms has been replicated four times on the same sample, and should extend to any setup using similar scanning devices. Investigation of the difference between classical TIRF, ASTER TIRF and spinning TIRF images has been realized three times.
Nanoruler PAINT experiments have been replicated three times for different illumination configurations, such as the one presented in the manuscript.
200 µm x 200 µm direct imaging in STORM has been repeated at least 8 times on different samples. Cluster analyses of clathrin has been performed on two samples, only one of which is shown in the manuscript. Wide FOV imaging of neurons has been repeated five times.
For comparison with ASTER, Gaussian illumination in STORM has been performed twice. Stitching experiment was replicated once. Concerning the ability to perform fast STORM imaging, it has been replicated consistently on two different setups and numerous samples (n>10).
Investigation of the effect of the scanning speed on STORM blinking kinetics has been performed once. 3D Images of microtubules on a 200 µm x 200 µm² FOV has been performed thrice.

Randomization

Our experiments were primarily concerned with development of new illumination tools and image analyses. Thus blinding and randomization is not applicable to our work.

Blinding

Our experiments were primarily concerned with development of new illumination tools and image analyses. Thus blinding and randomization is not applicable to our work.

Reporting for specific materials, systems and methods

We require information from authors about some types of materials, experimental systems and methods used in many studies. Here, indicate whether each material, system or method listed is relevant to your study. If you are not sure if a list item applies to your research, read the appropriate section before selecting a response.

Materials & experimental systems

n/a	Involved in the study	
[ ]	[x]	Antibodies
[ ]	[x]	Eukaryotic cell lines
[x]	[ ]	Palaeontology and archaeology
[ ]	[x]	Animals and other organisms
[x]	[ ]	Human research participants
[x]	[ ]	Clinical data
[x]	[ ]	Dual use research of concern

Methods

n/a	Involved in the study	
[x]	[ ]	ChIP-seq
[x]	[ ]	Flow cytometry
[x]	[ ]	MRI-based neuroimaging

Antibodies

Antibodies used

α-tubulin (Sigma Aldrich, T6199), clathrin heavy-chain (Abcam, ab2731) ,mouse anti β2-spectrin (BD Sciences, #612563)

Validation

All antibodys resulted in specific staining showing the expected structure as expected from manufacturers website.

Eukaryotic cell lines

Policy information about cell lines

Cell line source(s)	COS7 Cell lines were purchased through Cellulonet, a french center for biological ressource that follows NFS96-900 norms
Authentication	None of the cell lines used were authenticated by another institution to confirm Cellulonet shipment.
Mycoplasma contamination	Cell lines are negative to mycoplasma and are regurlarly tested to check for any contamination
Commonly misidentified lines (See ICLAC register)	no commonly misidetified cell lines

Animals and other organisms

Policy information about studies involving animals; ARRIVE guidelines recommended for reporting animal research

Laboratory animals	E18 Wistar rat embryo s, both sexes, were used for the hippocampal neuron culture.
Wild animals	No wild animals were used
Field-collected samples	no field collected samples were used
Ethics oversight	Neurons from E18 Wistar rat embryo hippocampi were studied. Protocols followed the guidelines from European Animal Care and Use Committee (86/609/CEE) and were approved by local ethics committee (agreement D13-055-8).

Note that full information on the approval of the study protocol must also be provided in the manuscript.
